# Progressive and Stable Synaptic Plasticity with Femtojoule Energy Consumption by the Interface Engineering of a Metal/Ferroelectric/Semiconductor

**DOI:** 10.1002/advs.202201502

**Published:** 2022-05-24

**Authors:** Sohwi Kim, Chansoo Yoon, Gwangtaek Oh, Young Woong Lee, Minjeong Shin, Eun Hee Kee, Bae Ho Park, Ji Hye Lee, Sanghyun Park, Bo Soo Kang, Young Heon Kim

**Affiliations:** ^1^ Division of Quantum Phases & Devices Department of Physics Konkuk University Seoul 05029 South Korea; ^2^ Center for Correlated Electron Systems (CCES) Institute of Basic Science (IBS) Seoul 08826 South Korea; ^3^ Department of Physics and Astronomy Seoul National University Seoul 08826 South Korea; ^4^ Department of Applied Physics Hanyang University Gyeonggi‐do 15588 South Korea; ^5^ Graduate School of Analytical Science and Technology Chungnam National University Daejoen 34134 South Korea

**Keywords:** energy efficiency, linearity, low reading current, short operation time, symmetry

## Abstract

In the era of “big data,” the cognitive system of the human brain is being mimicked through hardware implementation of highly accurate neuromorphic computing by progressive weight update in synaptic electronics. Low‐energy synaptic operation requires both low reading current and short operation time to be applicable to large‐scale neuromorphic computing systems. In this study, an energy‐efficient synaptic device is implemented comprising a Ni/Pb(Zr_0.52_Ti_0.48_)O_3_ (PZT)/0.5 wt.% Nb‐doped SrTiO_3_ (Nb:STO) heterojunction with a low reading current of 10 nA and short operation time of 20–100 ns. Ultralow femtojoule operation below 9 fJ at a synaptic event, which is comparable to the energy required for synaptic events in the human brain (10 fJ), is achieved by adjusting the Schottky barrier between the top electrode and ferroelectric film. Moreover, progressive domain switching in ferroelectric PZT successfully induces both low nonlinearity/asymmetry and good stability of the weight update. The synaptic device developed here can facilitate the development of large‐scale neuromorphic arrays for artificial neural networks with low energy consumption and high accuracy.

## Graphical abstract

In this study, an energy‐efficient synaptic device based on a Ni/Pb(Zr_0.52_Ti_0.48_)O_3_ (PZT)/Nb‐doped SrTiO_3_ heterojunction with a low reading current and a short operation time that results from the interface engineering between top electrode and PZT is implemented. Furthermore, PZT ferroelectric with multi‐domain configurations can enable electrical modulation gradually and stably, leading to linear/symmetric synaptic plasticity with small variability.

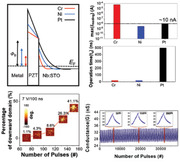

## 1 Introduction

The hardware implementation of neuromorphic computing requires the emulation of biological synapses whose plasticity provides the physiological substrate for a variety of neuromorphic computing and learning.^[^ ^1^ ^]^ The energy‐efficient and parallel execution of learning and inference in a large crossbar array over 1000 × 1000 synapses are desirable for an ideal neuromorphic system.^[^ ^1^ ^]^ Further, low energy consumption and high dot‐product computing accuracy require a reading current lower than 10 nA during weight updates.^[^ ^2^, ^3^ ^]^ The energy‐efficient storage and processing of massive amounts of information require a short operation time in the range of nanoseconds and low operation voltage.^[^ ^4^, ^5^, ^6^ ^]^ Symmetrically/linearly programmable conductance states under voltage training are also necessary for increasing the recognition accuracy in large‐scale neuromorphic arrays.^[^ ^1^, ^7^ ^]^

Two‐terminal memristors have attracted considerable attention in implementing synaptic devices owing to their structural and functional similarity with biological synapses and easy integration with the crossbar array.^[^ ^5^, ^8^ ^]^ Among the various two‐terminal memristors, metal/ferroelectric/semiconductor (MFS) memristors utilizing Nb‐doped SrTiO_3_ as a semiconducting substrate have exhibited high performance in resistive switching behaviors owing to the modulation of the ferroelectric barrier and depletion region in Nb‐doped SrTiO_3_ semiconductors by ferroelectric polarization reversal.^[^ ^9^, ^10^, ^11^, ^12^, ^13^, ^14^, ^15^ ^]^ Progressive polarization reversal of the ferroelectric domain in MFS can improve the electrical modulation of the band profile between the high resistance state (HRS) and low resistance state (LRS), which can lead to high linearity/symmetry of the synaptic weight update when using a smart programming pulse.^[^ ^9^, ^10^, ^11^, ^12^, ^13^, ^16^ ^]^ However, parameter enhancement in weight update (linearity, symmetry, and variation) caused by interface engineering between the ferroelectric material and top electrode has not been addressed in MFS memristors reported earlier.^[^ ^4^, ^8^, ^11^, ^14^, ^15^ ^]^ Moreover, high reading currents of the order of µA during synaptic operation pose a major challenge in implementing energy‐efficient large‐scale synaptic arrays based on MFS devices, although ultrafast synaptic operation with low energy consumption has been achieved.^[^ ^2^, ^3^, ^4^, ^8^, ^11^, ^14^, ^15^ ^]^

In this study, we fabricated a high‐performance single synaptic device based on a Ni/Pb(Zr_0.52_Ti_0.48_)O_3_ (PZT)/0.5 wt.% Nb‐doped SrTiO_3_ (Nb:STO) MFS structure to develop an energy‐efficient and accurate neuromorphic array. Engineering the interface barrier between the top electrode and PZT results in an ultralow reading current below 10 nA and a short operation time in the range of nanoseconds (20–100 ns). This lowers the minimum energy consumption in our device up to a few femtojoules or less at a pulse duration of 20 ns, which is significantly lower than those of biological synapses (10 fJ). In addition, the gradual electrical modulation and high stability owing to a ferroelectric layer in the Ni/PZT/Nb:STO device provide linear (nonlinearity factor of 0.01 for potentiation and 2.9 for depression) and symmetric (asymmetric ratio of 0.18) synaptic plasticity with a low relative standard deviation (0.9% for 300 cycles). Our synaptic device, characterized by femtojoule energy consumption and high linearity/symmetry, can be considered an essential building block of highly energy‐efficient, reliable, and accurate neuromorphic computing systems.

## 2 Results and Discussion

### 2.1 Structural and Ferroelectric Characterizations of PZT/Nb:STO

**Figure 1:**
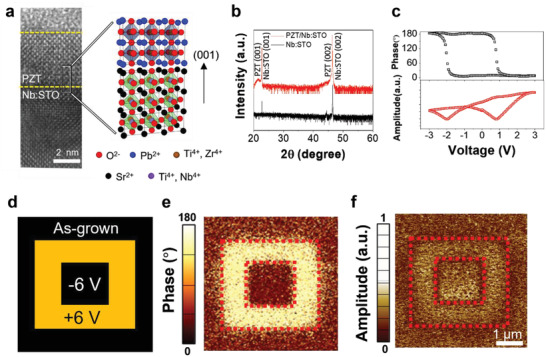
Properties of a PZT film deposited on Nb:STO substrate. a) Cross‐sectional high resolutionTEM image and schematic diagram of the PZT/Nb:STO interface with 6‐nm‐thick PZT film. b) XRD *θ*–2*θ* scan data of Nb:STO substrate and PZT/Nb:STO structure. c) Hysteretic behaviors of the out‐of‐plane PFM phase and amplitude signals measured on a fixed point of the bare PZT surface. d) External voltages applied for ferroelectric domain patterning on PZT surface. e,f) Out‐of‐plane PFM images of phase (e) and amplitude (f) acquired after the domain patterning on a PZT/Nb:STO structure.

Ferroelectric PZT films were grown on a single‐crystal Nb:STO substrate with a TiO_2_‐terminated step‐terrace surface using pulsed laser deposition (see Experimental Section). Nb:STO was selected as the substrate material because of its small lattice mismatch with PZT and excellent conductivity.^[^ ^17^ ^]^ **Figure** **1**a shows a cross‐sectional transmission electron microscopy (TEM) image and schematic diagram of the interface structure of the PZT/Nb:STO heterostructure with a 6 nm‐thick PZT layer. The X‐ray diffraction (XRD) data of the Nb:STO substrate and PZT/Nb:STO structure in Figure 1b shows the Bragg peaks for both the Nb:STO substrate and PZT film. The XRD pattern shows only PZT (00*l*) peaks, implying the preferential growth of the PZT film along the *c*‐axis normal to the substrate.

Figure 1c displays the hysteretic behaviors of the out‐of‐plane piezo‐response force microscopy (PFM) phase and amplitude, which are obtained at a specific point on bare PZT surface by sweeping the voltage from 3.0 V to −3.0 V (see Experimental Section). The coercive voltages, at which the phase changes abruptly, are determined as +1 V and −2 V. The ferroelectric behaviors of PZT films were also investigated by patterning ferroelectric domains on them with external voltages, as shown in Figure 1d. The out‐of‐plane PFM phase and amplitude images obtained after poling are shown in Figure 1e,f, respectively (see Experimental Section). The out‐of‐plane PFM phase image shows a 180° phase contrast and distinguishable domain boundary between the inversely poled areas. Furthermore, the ferroelectric domains with switchable remnant polarizations possess nearly the same amplitude, which indicates that antiparallel ferroelectric domains can be written in the PZT layer. The PFM images and hysteresis loops confirm the ferroelectric properties of the PZT film grown on the Nb:STO substrate.

### 2.2 Effect of the Top Electrode on Synaptic Behaviors

**Figure 2:**
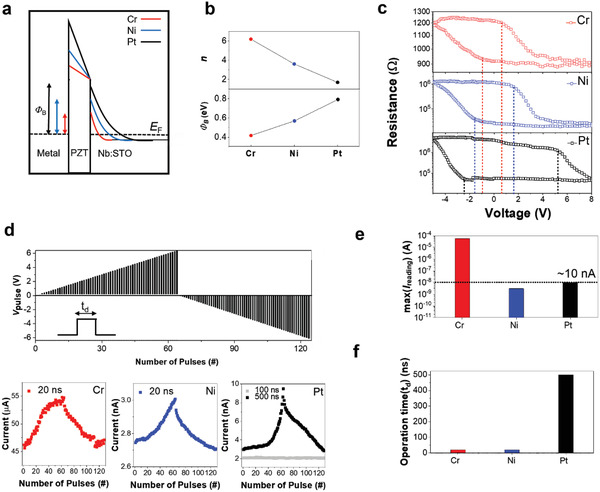
Optimization of top electrode for energy‐efficient synaptic performance in metal/PZT/Nb:STO. a,b) Band profiles (a) and junction parameters *n* and Φ_B_ at HRS (b) of metal/PZT/Nb:STO structures. c,d) Resistance (*R*)‐pulse voltage (*V*_p_) loops (c) and synaptic weight updates using a smart programming scheme (d) of the Cr/PZT/Nb:STO, Ni/PZT/Nb:STO, and Pt/PZT/Nb:STO. e,f) Maximum reading current (e) and operation time (f) during the synaptic weight updates of the Cr/PZT/Nb:STO, Ni/PZT/Nb:STO, and Pt/PZT/Nb:STO.

To implement large‐scale synaptic arrays with high neuromorphic accuracy and low energy consumption, low reading current under 10 nA, and nanosecond‐range operation time, even at a small external bias, should be simultaneously obtained by adjusting the barrier height at the metal/ferroelectric interface.^[^ ^2^, ^4^ ^]^ Considering the fixed electron affinity of Nb:STO (****χ****_Nb:STO_ = 3.9 eV), the Schottky barrier height (Φ_B_) in metal/PZT/Nb:STO (Figure S1, Supporting Information) can be controlled by the work function (Φ_metal_) of the metal top electrode according to Φ_B_∼ Φ_metal_−  *χ*_Nb:STO_ in semiconductor theory because the band bending at the PZT/Nb:STO interface is the function of Φ_metal_ and *χ*_Nb:STO_, as shown in **Figure** **2**a.^[^ ^4^, ^18^ ^]^ Thus, a suitable top electrode on the PZT film is essential for obtaining the optimum Φ_B_ in a metal/PZT/Nb:STO structure.

Cr, Ni, and Pt top electrodes with different *Φ*_metal_ values of 4.5, 5.4, and 5.7 eV, respectively, were deposited on the PZT film (see Experimental Section).^[^ ^17^ ^]^ Φ_B_ and the ideality factor (*n*) can be extracted from the equation of thermionic emission theory at a low forward bias:

(1) $$I\left(V\right)=SA^{*}T^{2}\exp\left(-\frac{q\Phi_{B}}{k_{B}T}\right)\left(\exp\left(\frac{qV}{nk_{B}T}\right)-1\right)$$

where *S* is the junction area, A*the Richardson constant, *T* the temperature, *k*_B_ the Boltzmann constant, and *q* the electron charge.^[^ ^19^, ^20^ ^]^ Typical current–voltage (*I–V*) sweep data for metal/PZT/Nb:STO with different Cr, Ni, and Pt top electrodes were obtained by applying an external voltage (Figure S2, Supporting Information). The HRS currents at low forward bias (≤0.5 V) were well fitted by equation (1), suggesting the dominant role of the Schottky barrier in the carrier transport.^[^ ^19^ ^]^ The junction parameters *n* and Φ_B_ of the HRS were estimated from the slope and intercept of the current axis in the ln(*I*)–*V* curve, respectively. *n* (Cr: 6.1, Ni: 3.6, Pt: 1.6) decreases while Φ_B_ (Cr: 0.41 eV, Ni: 0.56 eV, Pt: 0.79 eV) increases with increasing *Φ*_metal_ (Figure 2b).

Figure 2c shows the resistance (*R*)‐pulse voltage (*V*_p_) loops of the metal/PZT/Nb:STO devices with Cr, Ni, and Pt electrodes, which are obtained using pulses of 10 µs duration. *R* was measured at −0.1 V. A low operation voltage is sufficient for flipping the ferroelectric domains in the Cr/PZT/Nb:STO device with small Φ_B_. However, the large Φ_B_ in Pt/PZT/Nb:STO requires a large operation voltage to flip the ferroelectric domains. Because the total external voltage is partially applied to the PZT and Schottky barrier, a larger Φ_B_ decreases the partial voltage applied to the PZT layer.^[^ ^4^ ^]^ Therefore, a lower operation voltage at a given pulse duration is possible in the device with a smaller Φ_B_, resulting from a smaller *Φ*_metal_, which facilitates lower energy consumption.

A smart programming pulse scheme (64 multi‐states with amplitudes from 0.1 V to 6.4 V for potentiation with a step of 0.1 V; 64 multi‐states with amplitudes from −0.1 V to −6.4 V with a step of −0.1 V for depression; 10 ms duration and −0.1 V amplitude for read) was used to compare the characteristics of synaptic weight updates depending on the top electrode, as shown in Figure 2d. The maximum reading current (*I*_reading_) and operation time (*t*_d_) during synaptic modulation in the devices with various top electrodes are shown in Figure 2e,f. A small Φ_B_ in the Cr/PZT/Nb:STO device enables the flipping of ferroelectric polarization at a short *t*_d_ of 20 ns, which causes gradual synaptic weight update.^[^ ^4^ ^]^ However, a high *I*_reading_ of over 1 µA, caused by its small Φ_B_, may lead to high energy consumption in large‐scale synaptic arrays. In contrast, the large Φ_B_ in the Pt/PZT/Nb:STO device considerably reduces *I*_reading_ to under 10 nA, while it increases *t*_d_ up to 500 ns which is required for polarization reversal and synaptic weight update.^[^ ^4^ ^]^ Most importantly, Ni/PZT/Nb:STO with medium Φ_B_ shows a low *I*_reading_ under 10 nA and short *t*_d_ of 20 ns. Thus, the problem of high *I*_reading_ previously reported in MFS devices can be overcome by our Ni/PZT/Nb:STO device while maintaining a short operation time in the nanosecond range.^[^ ^8^, ^11^, ^14^, ^15^ ^]^

### 2.3 Effect of the Ferroelectric Film on Electrical Properties

We assume that carrier transport in metal/PZT/Nb:STO is governed by charge trapping/detrapping, as previously reported for metal/Nb:STO devices, which can be affected by barrier modulation at the PZT/Nb:STO interface by external voltage.^[^ ^20^, ^21^, ^22^, ^23^, ^24^, ^25^, ^26^ ^]^ The electrical characteristics based on charge trapping/detrapping in the Nb:STO substrate can be improved by inserting a ferroelectric film between the metal and Nb:STO substrate. The optimized Ni/PZT/Nb:STO synaptic device was employed to gain insight into the direct impact of the ferroelectric layer on synaptic performance. The basic electrical characteristics of the Ni/PZT/Nb:STO and Ni/Nb:STO devices were measured at room temperature. Bipolar resistive switching curves (counterclockwise) in both devices were repeatedly observed 500 times by sweeping the external voltage between −7.5 V and 2.5 V, while they exhibited different distributions of the HRS resistance, LRS resistance, and set voltage (Figure S3, Supporting Information). The HRS and LRS resistances of the Ni/PZT/Nb:STO device were more uniform than those of the Ni/Nb:STO device. Moreover, the Ni/PZT/Nb:STO device exhibited a narrow distribution of the set voltage (1.9%), which is comparable to those of single‐crystalline SiGe epitaxial random access memory (1%) and alloyed memristor for neuromorphic computing (3.3%).^[^ ^7^, ^27^ ^]^ The improved stability in the Ni/PZT/Nb:STO device is more suitable for the practical implementation of reliable and highly accurate neuromorphic computing systems.^[^ ^28^ ^]^ Device‐to‐device variation of set voltage for 50 Ni/PZT/Nb:STO devices was extracted with same voltage sweep, which shows small variation of ≈3% (Figure S4, Supporting Information).

**Figure 3:**
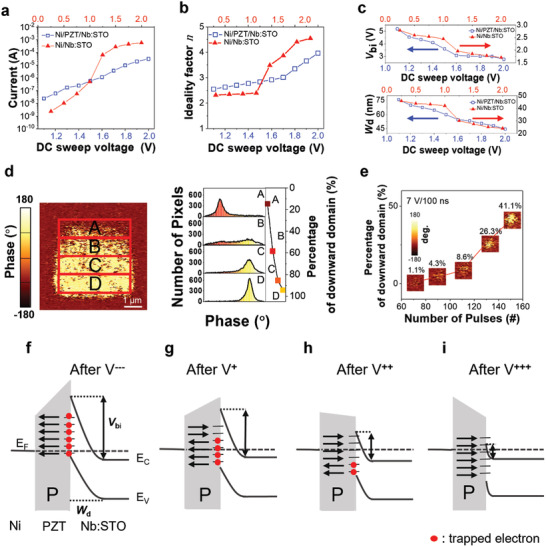
Gradual resistive switching based on progressive ferroelectric domain switching in Ni/PZT/Nb:STO. a–c) Current value (a), ideality factor (*n*) (b), built‐in potential (*V*_bi_), and depletion region width (*W*_d_) (c) of LRSs obtained by applying DC sweep voltages with different amplitudes to the Ni/PZT/Nb:STO and Ni/PZT/Nb:STO devices while maintaining the identical reset process. d) PFM phase images and domain distributions of various regions (A, B, C, and D) on PZT/Nb:STO, which are acquired after applying different positive DC voltages (0.5, 1.0, 2.0, and 3.0 V), respectively. e) Evolution of downward domain (bright color) as a function of the number of pulses with amplitude and duration of +7 V and 100 ns, respectively, which are applied at a fixed tip. f–i) Band profiles of Ni/PZT/Nb:STO devices at HRS (f) and LRSs obtained by applying different positive voltages (g–i). The black arrow and red circle denote the polarization direction and electron captured at the trap site, respectively.

**Figure** **3**a shows the current level (reading at 0.1 V) of the LRS as a function of the DC sweep voltage in the Ni/PZT/Nb:STO and Ni/Nb:STO devices, which are extracted from Figure S5 (Supporting Information). Different *I–V* curves were obtained under different DC sweep voltages while maintaining the same reset process. The LRS current was controlled by tuning the DC sweep voltage. In the Ni/PZT/Nb:STO, it increases gradually from 2.5 × 10^−8^ A to 3.2 × 10^−5^ A as the DC sweep voltage increases from 1.1 to 2.0 V in steps of 0.1 V. However, the LRS current in the Ni/Nb:STO device changes abruptly from 2.4 × 10^−9^ A to 5.9 × 10^−4^ A as the DC sweep voltage increases from 0.25 to 2.00 V in steps of 0.25 V. The gradual current modulation in the Ni/PZT/Nb:STO device benefits on‐chip training that relies on precise synaptic weight updates for rapid convergence.^[^ ^29^ ^]^ Furthermore, it can also provide a linear behavior of current update by pulse training, which determines the training accuracy in neuromorphic computing.^[^ ^30^ ^]^

Figure 3b shows the *n* value of the LRS as a function of the DC sweep voltage in Ni/PZT/Nb:STO and Ni/Nb:STO devices. It was estimated from the slope of the linear region of the ln*(J*_F_ *)–V* curve by using equation (1), where *J*_F_ represents the forward‐bias current density (Figure S6, Supporting Information). The *n* of the LRS increases with the amplitude of the DC sweep voltage in both devices. The *n* of LRS in the Ni/PZT/Nb:STO device increases gradually from 2.55 to 3.96 with increasing DC sweep voltage from 1.1 to 2.0 V in steps of 0.1 V. However, the *n* of LRS in the Ni/Nb:STO device changes more abruptly from 2.02 to 4.49 with increasing DC sweep voltage from 0.25 to 2.00 V in steps of 0.25 V.

In an MFS junction, the interfacial ferroelectric layer has a capacitance *C*_i_ and it can partition the applied voltage *V* with *V*_i_ = *V*(*n* − 1)/*n*. Consequently, the voltage drop across the depletion region is thus reduced to *V*_d_ = *V*/*n*. Here, *n* can be expressed as^[^ ^19^, ^20^ ^]^,

(2) $$n=1+\frac{C_{d}}{C_{i}}=1+\frac{d}{W_{d}}\cdot \frac{\varepsilon_{r}}{\varepsilon_{i}}$$

where *C*_d_ and *C*_i_ are the high‐frequency capacitances of the depletion region in the semiconductor and the insulator layer, respectively, *ε*_r_ and *ε*_i_ are their respective relative dielectric constants, *W*_d_ is the depletion region width, and *d* is the thickness of the insulator. Here, *ε*_r_ of Nb:STO was estimated to be 290 at room temperature.^[^ ^19^, ^31^ ^]^ An inversely proportional relationship between *n* and *W*_d_ indicates a gradual increase in *n* caused by a gradual decrease in *W*_d_, for a fixed thickness of the insulator layer. *W*_d_ was determined experimentally using the built‐in potential (*V*_bi_) value estimated from the *C*_d_^−2^–*V*_d_ curve (Figure S7, Supporting Information). The values of *V*_bi_ and *W*_d_, which depend on the DC sweep voltage, are presented in Figure 3c. The *V*_bi_ and *W*_d_ of the Ni/PZT/Nb:STO device decrease from 5.18 to 2.77 V and from 76 to 44 nm, respectively, when the DC sweep voltage increases from 1.1 to 2.0 V in steps of 0.1 V. In the Ni/Nb:STO device, *V*_bi_ and *W*_d_ decrease from 2.78 to 1.70 V and from 46 to 24 nm, respectively, when the DC sweep voltage increases from 0.25 to 2.00 V in steps of 0.25 V. The data in Figure 3a–c suggest that the gradual control of the current in the Ni/PZT/Nb:STO device is correlated with the progressive control of *n*, *V*_bi_, and *W*_d_, owing to the gradual change in the ferroelectric domain configuration and the resultant charge trapping/detrapping. However, the Ni/Nb:STO shows abrupt changes in *n*, *V*_i_, and *W*_d_ due to abrupt charge trapping/detrapping, which is induced by only external voltage without ferroelectric bound charges of PZT.

The PFM data in Figure 3d,e confirms the progressive modulation of the ferroelectric domain configuration by external bias. In Figure 3d, we first scanned a 6 × 6 *μ*m^2^ area with a DC bias of −10.5 V applied to the cantilever tip to orient the polarization in the upward direction. Then, we reversed the polarization direction by applying DC bias of 0.5, 1.0, 2.0, and 3.0 V to each 4 × 1 µm^2^ area of A, B, C, and D, respectively (see Experimental Section). Figure 3d also reveals the distribution of the downward domain after scanning. The number of pixels with downward domains increases with applied voltage. The percentages of downward domains for A, B, C, and D are 14.6%, 58.3%, 85.5%, and 94.3%, respectively.

Because synaptic weight was generally updated using a pulsed signal, we monitored the evolution of the out‐of‐plane PFM phase image by applying voltage pulses with a duration of 100 ns (Figure 3e).^[^ ^4^, ^32^ ^]^ We first scanned the 1 × 1 µm^2^ area on the PZT film by applying a DC bias of −9.0 V to the tip to create an upward ferroelectric domain. Then, we applied several positive pulses, whose amplitude and duration were 7.0 V and 100 ns, respectively, to a fixed tip on the PZT film to partially change the domain distribution. The percentage of the downward domain in the 1 × 1 µm^2^ area gradually increases with the number of pulses: 1.1%, 4.3%, 8.6%, 26.3%, and 41.1% for 70, 90, 110, 130, and 150 pulses, respectively. The evolution of the domain configuration in Figure 3d,e confirms the progressive reversal of the polarization from upward to downward, depending on the amplitude of the DC bias and number of voltage pulses.

Figure 3f–i shows the band profiles of the metal/PZT/Nb:STO device for HRS and LRS, which are determined by different ferroelectric domain distributions. When a large reverse (negative) bias is applied to the top electrode (Figure 3f), the negative bound charge in the PZT (upward polarization) increases the barrier at the PZT/Nb:STO interface. Electrons can be captured by the empty positively charged trap sites at the PZT/Nb:STO interface under reverse bias.^[^ ^33^ ^]^ The trapped charges increase both *V*_bi_ and *W*_d_ in Nb:STO, leading to HRS.^[^ ^20^ ^]^ After eliminating external bias, the trap sites can remain occupied owing to the large interface barrier caused by the upward remnant polarization in PZT. When a forward (positive) bias is applied to the top electrode (Figure 3g–i), the positive bound charge in PZT lowers the barrier at the PZT/Nb:STO interface. Trapped electrons are discharged from the trap states under forward bias.^[^ ^33^ ^]^ The detrapped states decrease both *V*_bi_ and *W*_d_ in Nb:STO, leading to LRS. Consequently, the gradual change in the distribution of trapped/detrapped states, which are induced by the domain evolution of ferroelectric remnant polarization, can progressively modulate the band profile, as shown in Figure 3f–i. The gradual multi‐domain configurations in a ferroelectric layer are expected to gradually change the band profiles at the PZT/Nb:STO interface and then induce more gradual multi‐level current than the device without PZT.

### 2.4 Effect of the Ferroelectric Film on Synaptic Performance

**Figure 4:**
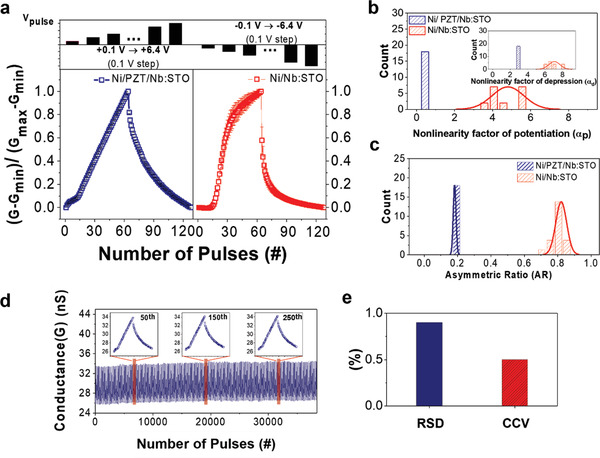
Linear and symmetric synaptic plasticity of Ni/PZT/Nb:STO a) Analog synaptic behaviors (18 potentiation/depression cycles) obtained using a smart programming scheme, with a pulse duration of 100 ns and 64 multi‐states, for Ni/PZT/Nb:STO and Ni/Nb:STO devices. b,c) Distribution of the nonlinearity factor of potentiation (*α*_*p*_) (b) and asymmetric ratio (AR) (c) for Ni/PZT/Nb:STO and Ni/Nb:STO devices. The inset of (b) shows the distribution of nonlinearity factor of depression (*α*_d_). d) Potentiation/depression behaviors of the Ni/PZT/Nb:STO during 300 cycles (38 400 pulses) under smart programming with 100 ns duration. The inset shows the potentiation/depression curves during the 50, 150, and 250th cycles. e) Relative standard deviation (RSD) of current values for the 300 cycles and CCV for the last cycle.

Synaptic devices with high linearity/symmetry are suitable for high‐accuracy neuromorphic computing systems and can constitute a transistor‐less memristor array with high scalability and stackability.^[^ ^6^, ^34^ ^]^ The linearity/symmetry of weight updates for high‐performance synaptic devices can be improved by applying a smart programming scheme.^[^ ^16^ ^]^ As shown in **Figure** **4**a, our smart programming scheme was applied to the Ni/PZT/Nb:STO device and Ni/Nb:STO. During 18 potentiation‐depression cycles, the Ni/PZT/Nb:STO device shows more linear/symmetric and stable analog weight updates than the Ni/Nb:STO device. We obtained temporal statistics of the nonlinearity factor and asymmetric ratio (AR) for 18 cycles for a more quantitative analysis (see Experimental Section and Figure S8, Supporting Information). The potentiation and depression curves were simulated using NeuroSim to determine the nonlinearity factor.^[^ ^35^ ^]^ Further, the relative standard deviation (RSD) in cyclic endurance tests, which is the ratio of the standard deviation (*σ*) to the mean (μ), was also estimated owing to the negative impact of device variability on the training and recognition process.^[^ ^36^ ^]^ Figure 4b shows that the average value and RSD for the nonlinearity factor of the Ni/PZT/Nb:STO device are 0.01 and *σ*_p_/μ_p_ = 0% for potentiation and 2.90 and *σ*_d_/μ_d_ = 0% for depression, respectively, whereas those of the Ni/Nb:STO device are 4.80 and *σ*_p_/μ_p_ = 17% for potentiation and 7.06 and *σ*_d_/μ_d_ = 9% for depression, respectively. The average value and RSD for AR of the Ni/PZT/Nb:STO device are 0.18 and *σ*/μ = 0.83%, whereas those of the Ni/Nb:STO device are 0.82 and *σ*/μ = 3.6%, respectively, as shown in Figure 4c. The linear and symmetric analog update with the incremental pulse scheme in the Ni/PZT/Nb:STO device is considered to originate from the multi‐domain response of PZT, where individual ferroelectric domains possess different coercive voltages and react differently by train of pulses.^[^ ^32^, ^34^ ^]^

High endurance is an important criterion for reliable crossbar arrays in neuromorphic computing.^[^ ^28^ ^]^ In particular, the endurance of conductance tuning is an important concern for high accuracy in situ training in array‐level implementation.^[^ ^9^, ^10^, ^37^ ^]^ In situ learning requires a network to continuously adapt and update its knowledge as more training data become available, which significantly improves accuracy.^[^ ^10^ ^]^ Figure 4d shows that the Ni/PZT/Nb:STO device retains similar potentiation/depression behaviors during 300 cycles (38 400 pulse trainings) with a pulse duration of 100 ns. This indicates good endurance without drastic changes in conductance states. Figure 4e shows the average RSD for each conductance state of potentiation/depression during 300 cycles (Figure 4d) and the weight update cycle‐to‐cycle variation (CCV) for the last cycle in our Ni/PZT/Nb:STO device (Figure S9, Supporting Information). CCV is distinct from RSD and is the variation in conductance change at every programming pulse, which influences the number of multi‐states and nonlinearity.^[^ ^35^, ^38^ ^]^ The CCV of synaptic modulation was studied using the NeuroSim simulator in our device.^[^ ^35^ ^]^ Our Ni/PZT/Nb:STO device displays an ultralow average RSD of 0.9% for 300 cycles. Recently, Pt/SnS/Pt and carrageenan/Ag nanocomposite devices showed small RSDs of 6.27% and 2.41% for 100 cycles, respectively, during smart programming.^[^ ^36^, ^39^ ^]^ An ultralow weight update CCV (<0.5%) in our Ni/PZT/Nb:STO device satisfies the targeted value of CCV (<2%) for high learning accuracy. Thus, the small RSD and CCV values imply the good stability of the synaptic behaviors of the Ni/PZT/Nb:STO device, which has the potential for reliable large‐scale synaptic arrays in neuromorphic computing.^[^ ^40^ ^]^ Analog synaptic behaviors obtained using an identical programming scheme are also measured for Ni/PZT/Nb:STO and Ni/Nb:STO devices, which also shows more enhanced properties of nonlinearity factor and RSD in Ni/PZT/Nb:STO device (Figure S10, Supporting Information). In addition, we measured the *I–t* curves for confirming the long‐term memory properties of multi‐states, which are obtained by pulse train with varying the pulse number in Ni/PZT/Nb:STO device (Figure S11, Supporting Information).

**Figure 5:**
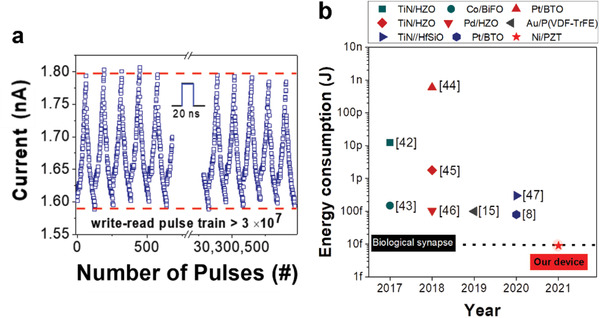
Femtojoule energy consumption with high endurance in Ni/PZT/Nb:STO. a) Demonstration of stable synaptic weight update with ultralow energy consumption below 9 fJ during over 3 × 10^7^ pulses. b) Statistical results of the reported two‐terminal synaptic memristors based on ferroelectric domain reversal in the recent five years.^[^ ^8^, ^15^, ^42^, ^43^, ^44^, ^45^, ^46^, ^47^ ^]^

In biological systems, the energy consumption per synaptic event is ≈10 fJ on average.^[^ ^5^ ^]^ Therefore, electronic synapses with low energy consumption (E = *V*_write_ × *I*_write_ × *t*) similar to that of biological operation are required for brain‐like neuromorphic computing.^[^ ^41^ ^]^ Repeated smart programming schemes over 3 × 10^7^ pulses with a duration of 20 ns were applied to the Ni/PZT/Nb:STO device, as shown in **Figure** **5**a. We achieved an extremely low energy consumption of below 9 fJ/bit for operation during the stimulations and good endurance over 3 × 10^7^ pulses. Our Ni/PZT/Nb:STO device exhibits an ultralow energy consumption below 9 fJ during weight updates, which is lower than that of biological synapses (<10 fJ) because of the 20 ns pulse duration and low reading current under 10 nA (Figure S12, Supporting Information). Figure 5b displays the statistical results of the energy consumption in two‐terminal synaptic devices modulated by ferroelectric domain switching over the last five years.^[^ ^8^, ^15^, ^42^, ^43^, ^44^, ^45^, ^46^, ^47^ ^]^ Notably, our Ni/PZT/Nb:STO device consumes the lowest energy during synaptic weight update among all the ferroelectric‐based two‐terminal synaptic devices. An appropriate top electrode is critical for reducing the operation time and reading current simultaneously, which are essential for low‐energy synaptic operation. The ultralow energy consumption (on the order of fJ) with high endurance in our Ni/PZT/Nb:STO device can extend neuromorphic computing to low‐power environments such as edge computing (e.g., mobile and wearable devices) or support adaptive neural networking.^[^ ^2^ ^]^

## 3 Conclusion

In conclusion, we implemented progressive and stable synaptic plasticity with femtojoule energy consumption in a synaptic device based on a MFS structure. Interface engineering between metals and ferroelectrics reduced the operation time and reading current required for ultralow energy consumption during synaptic weight updates. Ferroelectric thin films with multi‐domain configurations can enable progressive and stable changes in the distribution of trapped/detrapped states, which ensures linear/symmetric synaptic weight updates with small variability. Therefore, our Ni/PZT/Nb:STO device is a potential building block for reliable large‐scale synaptic arrays for high‐accuracy neuromorphic computing systems.

## 4 Experimental Section

### Device Fabrication

PZT films with a thickness of 6 nm were epitaxially grown on (001) single‐crystalline Nb:STO substrates (Figure S13, Supporting Information) by pulsed laser deposition using a KrF excimer laser (Coherent, COMPexPro 205F). Before deposition, the Nb:STO substrates were etched by NH_4_F buffered‐HF solution and annealed at 1000 °C for 1 h in air to form a TiO_2_‐terminated step‐terrace surface. The PZT films were deposited by using a laser energy density of 0.69 Jcm^−2^ at a repetition rate of 1 Hz, maintaining the substrate temperature and O_2_ pressure at 550 °C and 200 mTorr, respectively. Au/Cr (thickness of 25/30 nm), Au/Ni (thickness of 25/30 nm), and Pt (thickness of 50 nm) top electrodes with 20 × 20 µm^2^ in size were deposited and patterned on the surfaces of PZT/Nb:STO heterostructures by using e‐beam evaporation and lift‐off processes.

### Structural Characteristics

Cross‐sectional TEM images were obtained using a 300 kV field‐emission TEM (Tecnai G2 F30 super‐twin). The thicknesses of the films were calibrated and their structural characteristics were measured using TEM images.

### Ferroelectric Characterization

PFM images of the PZT/Nb:STO heterostructure were obtained using a Pt/Ir‐coated tip as the top electrode in a commercial atomic force microscope (Park systems, XE‐100). The local hysteresis loops and ferroelectric domain evolution were measured using a diamond‐coated conductive tip. A lock‐in amplifier (Stanford Research Systems, SR830) was used to apply an AC bias (f = 17 kHz) with an amplitude of 1.0 V (peak to peak) in the PFM mode.

### Electrical Measurements

The *I–V* characteristics in DC mode and gradual current modulation in pulse mode were measured using a probe station equipped with a semiconductor characterization system (Keithley, 4200‐SCS). The shortest pulse generated by the ultrafast module had a duration of 20 ns. The *C*–*V* characteristics were measured using an impedance analyzer (Agilent, 4294A) at room temperature with a frequency of 1 MHz and an oscillation level of 50 mV. All electrical measurements were performed at room temperature in air with 2 µm W probes (MS TECH, M1.5BT).

### Analysis of Analog Weight Updates

To analyze the impact of nonlinear weight updates on learning, the conductance change of potentiation (*G*_P_) and depression (*G*_D_) with the number of pulses (*P*) were described by the following equations:

(3) $$G_{P}=B\left(1-e^{\left(\frac{-P}{A}\right)}\right)+G_{\min}$$

(4) $$G_{D}=-B\left(1-e^{\left(\frac{P-P_{\max}}{A}\right)}\right)+G_{\max}$$

(5) $$B=\frac{G_{\max}-G_{\min}}{1-e^{\left(\frac{-P_{\max}}{A}\right)}}$$

where *G*_max_, *G*_min_, and *P*_max_, which are directly extracted from the experimental data, represent the maximum conductance, minimum conductance, and maximum number of pulses required to switch the device between the minimum and maximum conductance states, respectively.^[^ ^48^ ^]^ The parameter *A* controls the nonlinear behavior of the weight update, while *B* is simply a function of *A* that fits the functions within the range of *G*_max_, *G*_min_, and *P*_max_. *A* and *B* may be different in Equations (3) and (4). The nonlinearity factor *α* is defined corresponding to each A, as shown in Figure S8 (Supporting Information).^[^ ^35^ ^]^

Asymmetry indicates the degree of difference in the change of a certain conductance level between the potentiation and depression stages.^[^ ^28^ ^]^ It is also an important factor in high‐accuracy neuromorphic computing. The asymmetric ratio (AR) is defined as

(6) $$\mathrm{AR}=\frac{\max\left|G_{P}\left(n\right)-G_{D}\left(n_{\max}-n\right)\right|}{G_{P}\left(n_{\max}\right)-G_{D}\left(n_{\max}\right)}$$

where *G*_P_(n_max_) and *G*_D_(n_max_) are the channel conductance values obtained after applying the last potentiation and depression pulses, respectively, while *G*_P_(n) and *G*_D_(n_max_ − n) are the channel conductance values obtained after applying the n‐th potentiation and (n_max_ − n)‐th depression pulses, respectively. For the ideal symmetric case, AR is zero.^[^ ^49^ ^]^

### Statistical Analysis

The device conductance was calculated by the equation: *G* = *I*/*V*. The Origin software was used for data processing and analysis. Data variation such as CCV, RSD, and set voltage distribution was performed by Origin software and Excel software for the data processing and analysis.

## Conflict of Interest

The authors declare no conflict of interest.

## Supporting information

Supporting Information

## Data Availability

The data that support the findings of this study are available from the corresponding author upon reasonable request.

## References

[1] Y. Burgt , A. Melianas , S. Keene , G. Malliaras , A. Salleo , Nat. Electron. 2018, 1, 386.

[2] E. Fuller , S. Keene , A. Melianas , Z. Wang , S. Agarwal , Y. Li , Y. Tuchman , C. James , M. Marinella , J. Yang , A. Salleo , A. Talin , Science 2019, 364, 570. 31023890 10.1126/science.aaw5581

[3] S. Chen , R. Mahmoodi , Y. Shi , C. Mahata , B. Yuan , X. Liang , C. Wen , F. Hui , D. Akinwande , D. Strukov , M. Lanza , Nat. Electron. 2020, 3, 638.

[4] C. Ma , Z. Luo , W. Huang , L. Zhao , Q. Chen , Y. Lin , X. Liu , Z. Chen , C. Liu , H. Sun , X. Jin , Y. Yin , X. Li , Nat. Commun. 2020, 11, 1439. 32188861 10.1038/s41467-020-15249-1 PMC7080735

[5] D. Kuzum , S. Yu , H. Wong , Nanotechnology 2013, 24, 382001. 23999572 10.1088/0957-4484/24/38/382001

[6] P. Chen , X. Peng , S. Yu , IEEE Trans. Comput. Des. Integr. Circuits Syst. 2018, 37, 3067.

[7] S. Choi , S. Tan , Z. Li1 , Y. Kim , C. Choi , P. Chen , H. Yeon , S. Yu , J. Kim , Nat. Mater. 2018, 17, 335. 29358642 10.1038/s41563-017-0001-5

[8] J. Li , C. Ge , J. Du , C. Wang , G. Yang , K. Jin , Adv. Mater. 2020, 32, 1905764. 10.1002/adma.201905764 31850652

[9] A. Chanthbouala , V. Garcia1 , R. Cherifi , K. Bouzehouane , S. Fusil , X. Moya , S. Xavier , H. Yamada , C. Deranlot , N. Mathur , M. Bibes , A. Barthélémy , J. Grollier , Nat. Mater. 2012, 11, 860. 22983431 10.1038/nmat3415

[10] Z. Hu , Q. Li , M. Li , Q. Wang , Y. Zhu , X. Liu , X. Zhao , Y. Liu , S. Dong , Appl. Phys. Lett. 2013, 102, 102901.

[11] Z. Wen , D. Wu , A. Li , Appl. Phys. Lett. 2014, 105, 052910.

[12] V. Garcia1 , M. Bibes , Nat. Commun. 2014, 5, 4289. 25056141 10.1038/ncomms5289

[13] D. Kim , H. Lu , S. Ryu , C. Bark , C. Eom , E. Tsymbal , A. Gruverman , Nano Lett. 2012, 12, 5697. 23039785 10.1021/nl302912t

[14] G. Kolhatkar , B. Mittermeier , Y. Gonzalez , F. Weismueller , A. Sarkissian , C. Gomez‐Yanez , R. Thomas , C. Schindler , A. Ruediger , ACS Appl. Electron. Mater. 2019, 1, 828.

[15] S. Majumdar , Nanoscale 2021, 13, 11270. 34156059 10.1039/d1nr01722e

[16] Y. Chen , L. Gao , S. Yu , IEEE Trans. Multi‐Scale Comput. Syst. 2016, 2, 257.

[17] L. Skriver , N. Rosengaard , Phys. Rev. B 1992, 46, 7157. 10.1103/physrevb.46.7157 10002423

[18] Q. Gao , B. Chen , Q. Yu , X. Zhang , H. Zhu , J. Alloys Compd. 2013, 569, 62.

[19] Z. Xi , J. Ruan , C. Li , C. Zheng , Z. Wen , J. Dai , A. Li , D. Wu , Nat. Commun. 2017, 8, 15217. 28513590 10.1038/ncomms15217 PMC5442322

[20] E. Mikheev , B. Hoskins , D. Strukov , S. Stemmer , Nat. Commun. 2014, 5, 3990. 24886761 10.1038/ncomms4990 PMC4059928

[21] C. Park , Y. Seo , J. Jung , D. Kim , J. Appl. Phys. 2008, 103, 054106.

[22] E. Bourim , Y. Kim , D. Kim , ECS J. Solid State Sci. Technol. 2014, 3, N95.

[23] Z Yan , J. Liu , Sci. Rep. 2013, 3, 2482. 23963467 10.1038/srep02482 PMC3748856

[24] T. Fujii , M. Kawasaki , A. Sawa , H. Akoh , Appl. Phys. Lett. 2005, 86, 012107.

[25] X. Chen , X. Ma , Y. Yang , L. Chen , G. Xiong , G. Lian , Y. Yang , J. Yang , Appl. Phys. Lett. 2011, 98, 122102.

[26] M. Gwon , E. Lee , A. Sohn , J. Korean Phys. Soc. 2010, 57, 1432.

[27] H. Yeon , P. Lin , C. Choi , S. Tan , Y. Park , D. Lee , J. Lee , F. Xu , B. Gao , H. Wu , H. Qian , Y. Nie , S. Kim , J. Kim , Nat. Nanotechnol. 2020, 15, 574. 32514010 10.1038/s41565-020-0694-5

[28] M. Zhao , B. Gao , J. Tang , H. Qian , H. Wua , Appl. Phys. Rev. 2020, 7, 011301.

[29] T. Schranghamer , A. Oberoi , S. Das , Nat. Commun. 2020, 11, 5474. 33122647 10.1038/s41467-020-19203-z PMC7596564

[30] J. Bae , S. Lim , B. Park , J. Lee , IEEE Electron Device Lett. 2017, 38, 1153.

[31] C. Card , E. Rhoderick , J. Phys. D 1971, 4, 1589.

[32] M. Jerry , P. Chen , J. Zhang , P. Sharma , K. Ni , S. Yu , S. Datta , IEEE Int. Electron Devices Meet. 2017, 6.2.1.

[33] D. Kan , Y. Shimakawa , Appl. Phys. Lett. 2013, 103, 142910.

[34] M. Kim , J. Lee , Nano Lett. 2019, 19, 2044. 30698976 10.1021/acs.nanolett.9b00180

[35] P. Chen , X. Peng , S. Yu , IEEE Int. Electron Devices Meet. 2017, 6.1.1.

[36] K. Kwon , Y. Zhang , L. Wang , W. Yu , X. Wang , I. Park , H. Choi , T. Ma , Z. Zhu , B. Tian , C. Su , K. Loh , ACS Nano 2020, 14, 7628. 32492337 10.1021/acsnano.0c03869

[37] C. Li , D. Belkin , Y. Li , P. Yan , M. Hu , N. Ge , H. Jiang , E. Montgomery , P. Lin , Z. Wang , W. Song , J. Strachan , M. Barnell , Q. Wu , R. Williams , J. Yang , Q. Xia , Nat. Commun. 2018, 9, 2385. 29921923 10.1038/s41467-018-04484-2 PMC6008303

[38] S. Choi , J. Yang , G. Wang , Adv. Mater. 2020, 32, 2004659. 10.1002/adma.202004659 33006204

[39] J. Ge , D. Li , C. Huang , X. Zhao , J. Qin , H. Liu , W. Ye , W. Xu , Z. Liu , S. Pan , Nanoscale 2020, 12, 720. 31829372 10.1039/c9nr08001e

[40] S. Tan , P. Lin , H. Yeon , S. Choi , Y. Park , J. Kim , APL Mater. 2018, 6, 120901.

[41] C. Zhang , S. Wang , X. Zhao , Y. Yang , Y. Tong , M. Zhang , Q. Tang , Y. Liu , Adv. Funct. Mater. 2020, 31, 2007894.

[42] S. Oh , T. Kim , M. Kwak , J. Song , J. Woo , S. Jeon , I. Yoo , H. Hwang , IEEE Electron Device Lett. 2017, 38, 732.

[43] S. Boyn , J. Grollier , G. Lecerf , B. Xu , N. Locatelli , S. Fusil , S. Girod , C. Carrétéro , K. Garcia , S. Xavier , J. Tomas , L. Bellaiche , M. Bibes , A. Barthélémy , S. Saïghi , V. Garcia , Nat. Commun. 2017, 8, 14736. 28368007 10.1038/ncomms14736 PMC5382254

[44] R. Guo , Y. Zhou , L. Wu , Z. Wang , Z. Lim , X. Yan , W. Lin , H. Wang , H. Yoong , S. Chen , T. Ariando Venkatesan , J. Wang , G. Chow , A. Gruverman , X. Miao , Y. Zhu , J. Chen , ACS Appl. Mater. Interfaces 2018, 10, 12862. 29617112 10.1021/acsami.8b01469

[45] L. Chen , T. Wang , Y. Dai , M. Cha , H. Zhu , Q. Sun , S. Ding , P. Zhou , L. Chua , D. Zhang , Nanoscale 2018, 10, 15826. 30105324 10.1039/c8nr04734k

[46] H. Yoong , H. Wu , J. Zhao , H. Wang , R. Guo , J. Xiao , B. Zhang , P. Yang , S. Pennycook , N. Deng , X. Yan , J. Chen , Adv. Funct. Mater. 2018, 28, 1806037.

[47] R. Berdan , T. Marukame , K. Ota , M. Yamaguchi , M. Saitoh , S. Fujii , J. Deguchi , Y. Nishi , Nat. Electron. 2020, 3, 259.

[48] P. Chen , B. Lin , I. Wang , T. Hou , J. Ye , S. Vrudhula , J. Seo , Y. Cao , S. Yu , presented at 34th Proc. IEEE/ACM Int. Conf. Computer‐Aided Design, Austin, USA, Nov 2015.

[49] C. Yang , D. Shang , N. Liu , E. Fuller , S. Agrawal , A. Talin , Y. Li , B. Shen , Y. Sun , Adv. Funct. Mater. 2018, 28, 1804170.

