# Supplementary material for: Progressive and Stable Synaptic Plasticity with Femtojoule Energy Consumption by the Interface Engineering of a Metal/Ferroelectric/Semiconductor
Source: Adv Sci (Weinh). 2022 May 24;9(22):2201502. doi: 10.1002/advs.202201502 (PMC9353489; doi:10.1002/advs.202201502)
Supplement: Supplementary file 1 — Supporting Information [file ADVS-9-2201502-s001.pdf]

ADVANCED SCIENCE
Open Access

Supporting Information

for *Adv. Sci.*, DOI 10.1002/advs.202201502

Progressive and Stable Synaptic Plasticity with Femtojoule Energy Consumption by the Interface Engineering of a Metal/Ferroelectric/Semiconductor

Sohwi Kim, Chansoo Yoon, Gwangtaek Oh, Young Woong Lee, Minjeong Shin, Eun Hee Kee, Bae Ho Park, Ji Hye Lee, Sanghyun Park, Bo Soo Kang and Young Heon Kim*

Supporting Information

Progressive and stable synaptic plasticity with femtojoule energy consumption by the interface engineering of a metal/ferroelectric/semiconductor

Sohwi Kim, Chansoo Yoon, Gwangtaek Oh, Young Woong Lee, Minjeong Shin, Eun Hee Kee, Bae Ho Park, Ji Hye Lee, Sanghyun Park, Bo Soo Kang, and Young Heon Kim*

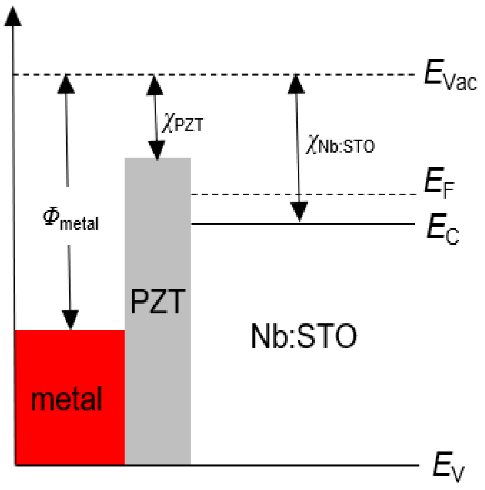

Figure S1. **Band profiles of the separated metal, PZT, and Nb:STO.**

Figure S1 depicts the work function of metal (Φ_{metal}: 4.5 eV (Cr), 5.35 eV (Ni), 5.7 eV (Pt)), electron affinity of PZT (χ_{PZT} = 3.5 eV), electron affinity of Nb:STO ($\chi_{Nb:STO}$ = 3.9 eV), vacuum level (E_{Vac}), conduction band minimum (E_C), valence band maximum (E_V), and Fermi level (E_F) of Nb:STO.[1–3] When the metal/PZT/Nb:STO junction is formed and reaches equilibrium, a Schottky barrier is formed. The Schottky barrier height (Φ_B) in a metal/PZT/Nb:STO can be controlled by the work function (Φ_{metal}) of the metal top electrode according to $\Phi_B \sim \Phi_{metal} - \chi_{Nb:STO}$ in semiconductor theory. The total external voltage applied to the metal/PZT/Nb:STO is shared by the PZT layer and Schottky barrier. The Schottky barrier is large when the Φ_{metal} of the metal electrode is large, which results in a small voltage drop across the PZT layer. Therefore, the PZT polarization domain can be reversed by a larger (smaller) voltage when a metal electrode with a larger (smaller) Φ_{metal} is used, and a larger Φ_B is induced.

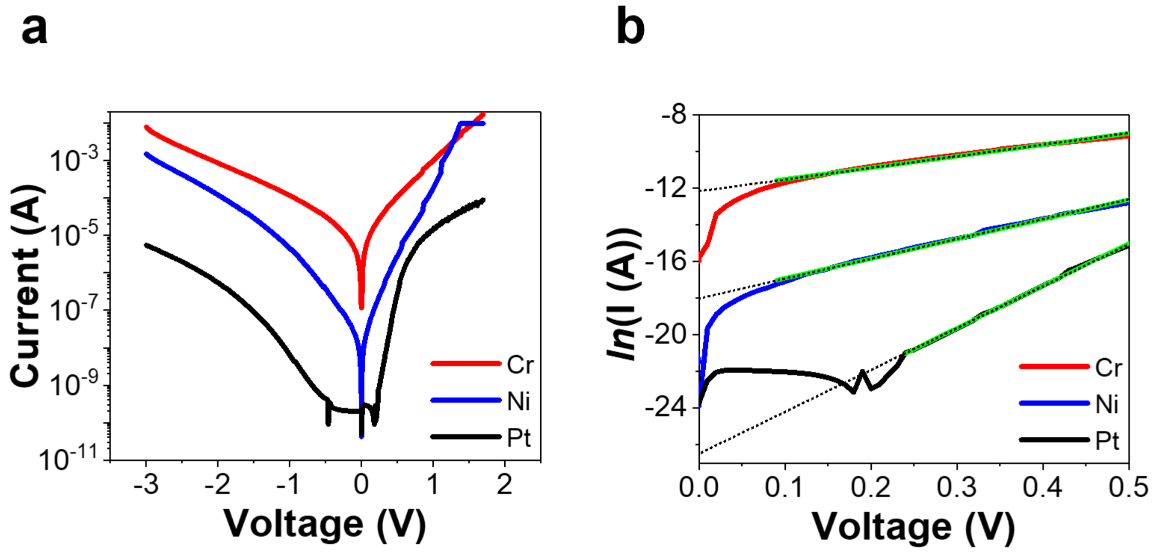

Figure S2. ***I*-*V* curves for metal/PZT/Nb:STO devices with different top electrodes.**

Figure S2a shows the HRS currents of metal/PZT/Nb:STO devices, which are acquired by sweeping the DC sweep from -3.0 to +1.7 V in steps of 0.02 V to the metal top electrode. The device, which has a metal top electrode with smaller (larger) Φ_{metal}, exhibits a higher (lower) current level. Figure S2b shows the ln(*I*)–*V* curves of metal/PZT/Nb:STO devices at HRS, which were extracted from Figure S2a. The dashed lines represent the fitting results based on the thermionic emission theory of ln(*I*)–*V* curves in the low forward bias region (< 0.5 V). Φ_B and the ideality factor (*n*) can be determined from the y-axis intercept and slope of each fitting line, respectively.

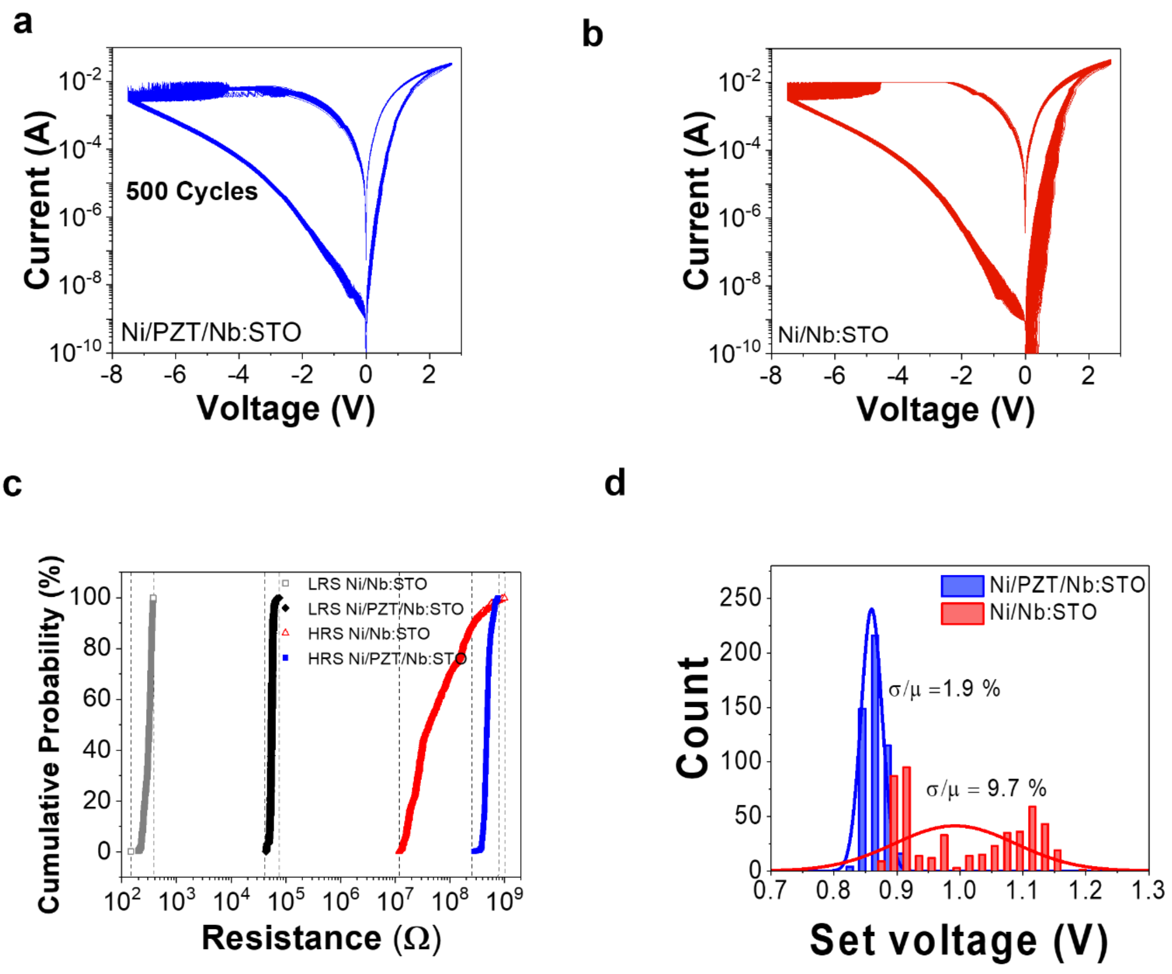

Figure S3. Stabilities of the HRS current, LRS current, and set voltage for the bipolar resistive switching of Ni/PZT/Nb:STO and Ni/Nb:STO.

As shown in Figure S3a and S3b, 500 bipolar switching curves of Ni/PZT/Nb:STO and NiNb:STO devices were obtained by repeatedly sweeping the external voltage between −7.5 V and 2.5 V with a step of 0.02 V. The two devices exhibit different stabilities for bipolar resistive switching. A more quantitative comparison was performed by measuring the cumulative probability of the HRS and LRS resistances (read at −1 V) of the two devices, as shown in Figure S3c. Both the HRS and LRS resistances of the Ni/PZT/Nb:STO device are much more uniform than those of the Ni/Nb:STO device. The set voltage distribution is shown in Figure S3d. The set voltage is defined as the voltage at which the current level reaches 300 μA during the set process.[4] The set voltage distribution is quantitatively

compared by introducing σ/μ, which is the ratio of the standard deviation (σ) to the mean value (μ) of the set voltage. A narrow set voltage distribution is important for accurate pattern learning/recognition in neuromorphic hardware.[4]

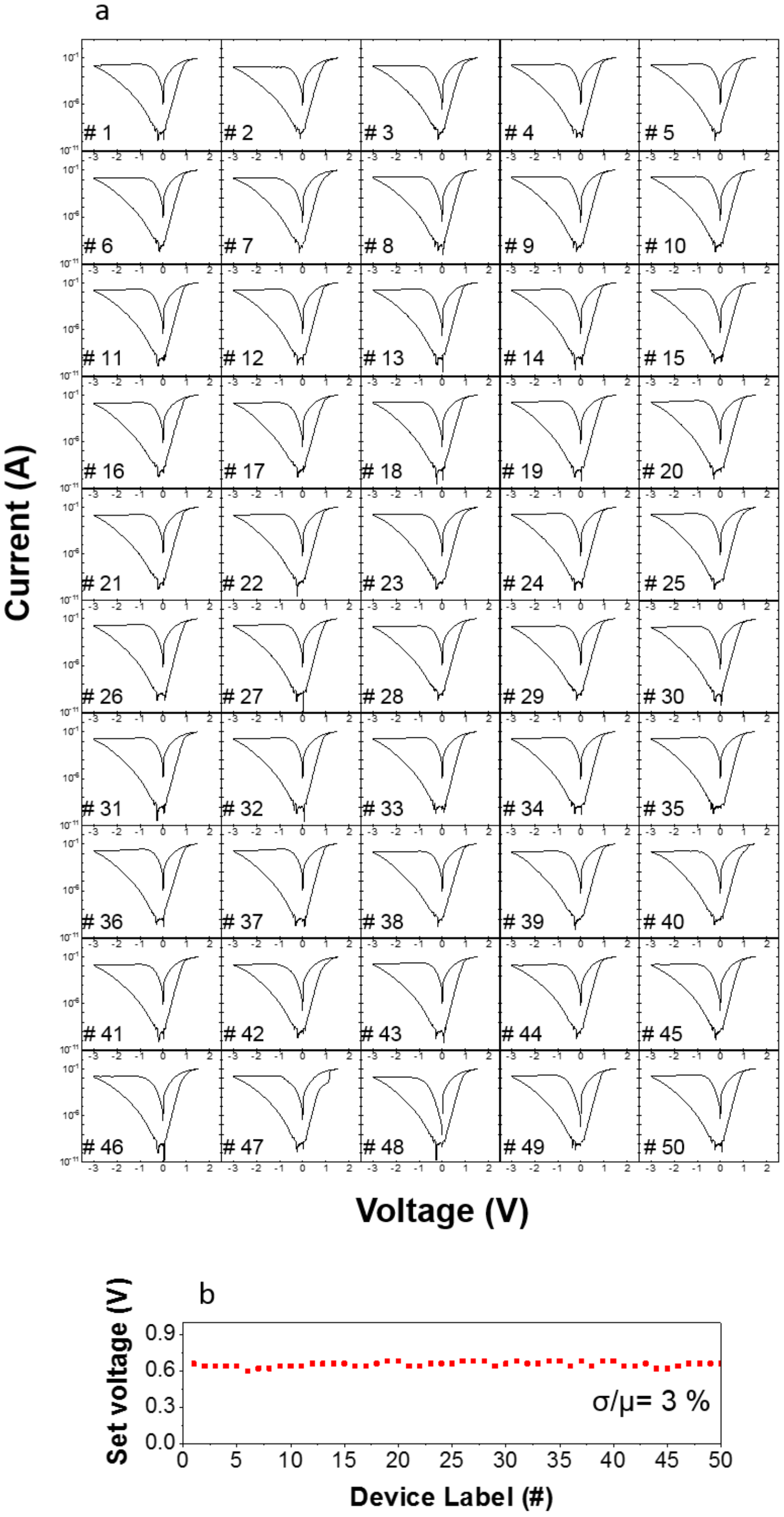

Figure S4. ***I-V*** **curves of 50 Ni/PZT/Nb:STO devices and device-to-device variation of set voltage.**

In order to measure the device-to-device variation of set voltage, we measured *I-V* curves of 50 Ni/PZT/Nb:STO devices. The set voltage is defined as the voltage at which the current level reaches 300 μA during the set process.[4] The device-to-device variation of set voltage is quantitatively evaluated by σ/μ which is the ratio of the standard deviation (σ) to the mean value (μ) of the set voltage. The Ni/PZT/Nb:STO device exhibits a small device-to-device variation of the set voltage of 3 %.

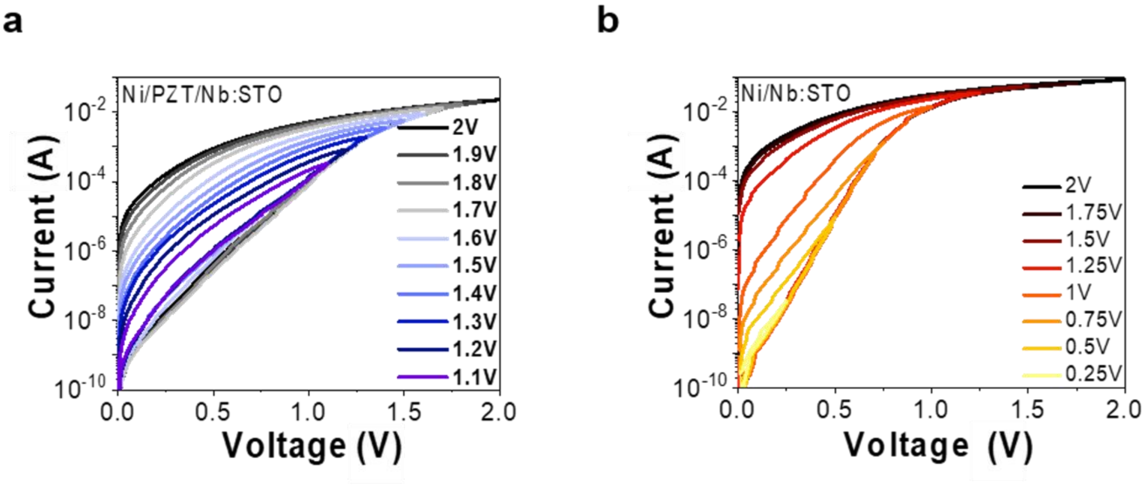

Figure S5. **Multi-level LRS currents obtained by applying different DC sweep voltages to the Ni/PZT/Nb:STO and Ni/Nb:STO.**

Figure S5 shows the different LRS current levels in the Ni/PZT/Nb:STO and Ni/Nb:STO devices, which were obtained by applying different DC sweep voltages to mimic analog synaptic behavior.[5] The multi-level currents of each device was achieved by controlling the charge trapping/detrapping states at the Nb:STO interface and the resultant engineering of the band structure owing to the modulation of the set voltage. Although the current level of the Ni/PZT/Nb:STO device changes gradually with increasing DC sweep voltage, that of Ni/Nb:STO changes abruptly, especially from 1.00 V to 1.25 V.

.

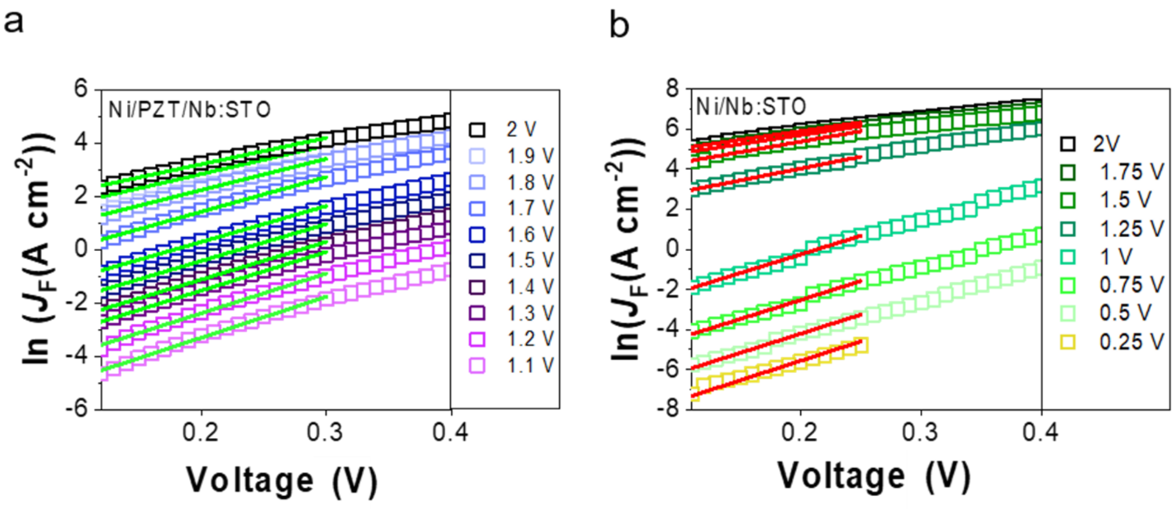

Figure S6. Linear fitting of the ln (J_F)-V curves of LRSs obtained using different DC sweep voltages for Ni/PZT/Nb:STO and Ni/Nb:STO.

The ideality factor n can be extracted from the slope of the linear region of the ln (J_F)-V curve in Figure S6 for Ni/PZT/Nb:STO and Ni/Nb:STO, whose top electrode size is 20 μm × 20 μm. The green and red lines in Figure S6a and S6b present the fitting results based on thermionic emission theory for the LRSs of the respective devices. All the ln (J_F)-V curves for the Ni/PZT/Nb:STO and Ni/Nb:STO devices are well fitted to the thermionic emission theory under 0.30 V and 0.25 V, respectively, suggesting the dominant role of the Schottky barrier in the carrier transport of both devices.[6]

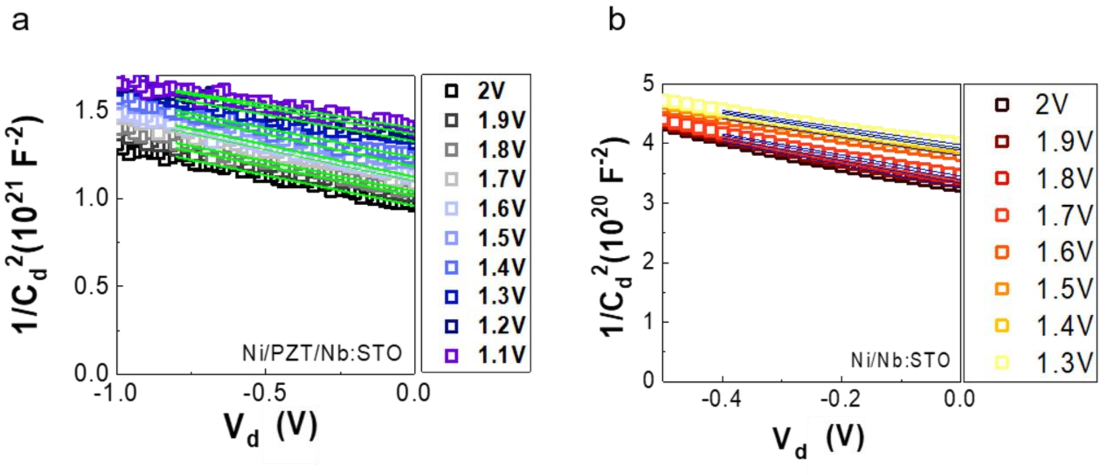

Figure S7. C_d^{-2}**-**V_d **characteristics of Ni/PZT/Nb:STO and Ni/Nb:STO.**

C_d^{-2}-V_d plots for the LRSs are obtained after applying different DC sweep voltages from +1.1 V +2.0 V with steps of +0.1 V (from +0.25 V to +2.00 V with steps of +0.25 V) to the Ni/PZT/Nb:STO (Ni/Nb:STO) device (Figure S7). The green (blue) solid lines show the fitting results based on the equation $C_d^{-2} = \frac{2(V_{bi}-V_d)}{q\varepsilon_0\varepsilon_r N_d}$, where C_d and V_d are expressed as nC and V/n, respectively, owing to the presence of the insulator layer.[6] The built-in potential (V_{bi}) and doping concentration N_d (~10^{19} cm^{-3}) of the Nb:STO substrate were extracted from the x-axis intercept and slope of the fitting curve, respectively. The width of the depletion region (W_d) was determined from the equation $W_d = \left(\frac{2\varepsilon_0\varepsilon_r V_{bi}}{qN_d}\right)^{\frac{1}{2}}$.

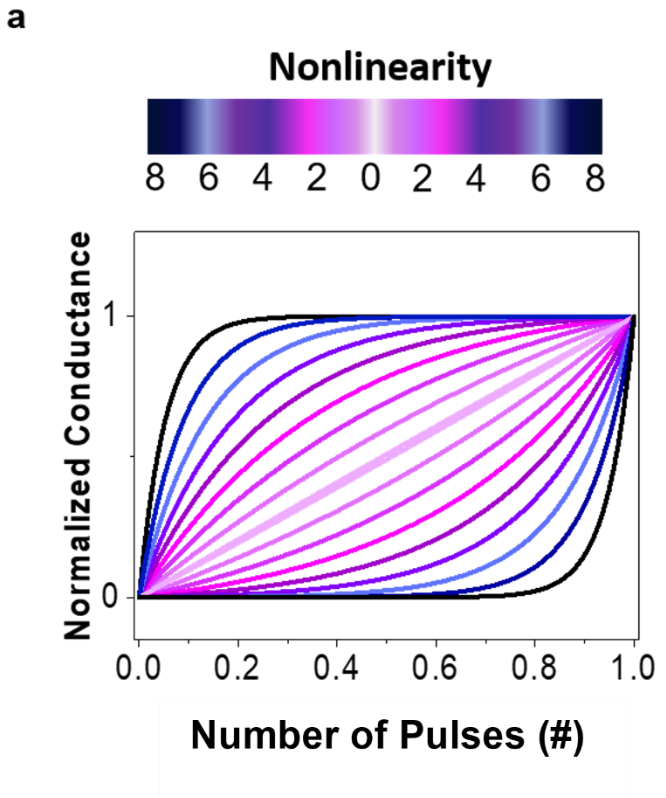

b

$A_{p,d}$	Nonlinearity($\alpha_{p,d}$)
±126.269	0.01
±1.2517	1
±0.609	2
±0.3868	3
±0.2691	4
±0.1924	5
±0.1356	6
±0.0901	7
±0.0528	8

Figure S8. Nonlinearity fittings of potentiation/depression in a normalized scale and definitions of nonlinearity factor ($\alpha_{p,d}$) corresponding to each A $_{p,d}$.

A linear change in the current update is closely related to pattern recognition accuracy in neuromorphic computing.[7,8] A linear conductance change during the potentiation and depression processes is desired in the performance of synaptic devices.[7] If the conductance state varies nonlinearly, additional periphery circuits such as transistors are typically required to manage a wide range of conductance state changes.[4] A set of nonlinear potentiation and depression behaviors can be obtained by adjusting *A*, as illustrated in Figure S8a, where each nonlinear curve is labeled by a nonlinearity value (α_p, α_d) from +8 to −8 in Figure S8b. The nonlinearity factor α was determined corresponding to each A, and more details are available in the literature.[9] The nonlinearity values should be zero for the ideal linear case.

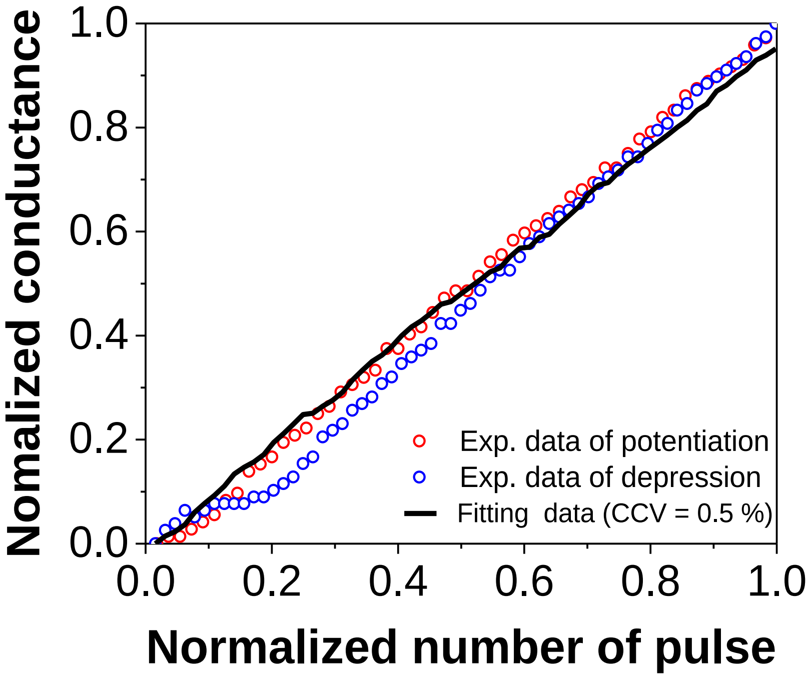

Figure S9. CCV of the synaptic devices.

The cycle-to-cycle variation (CCV) can influence the number of multistates and nonlinearity.[10] Normalizing the total number of pulses during weight updates to 1 by default definition, we can tune the CCV of the weight update for potentiation and depression to find the best fit, as shown in Figure S9. Our Ni/PZT/Nb:STO device exhibits an ultralow CCV of 0.5 % in weight update, which is significantly lower than the targeted value of CCV (< 2 %) required for high learning accuracy in an ideal synaptic device.[9]

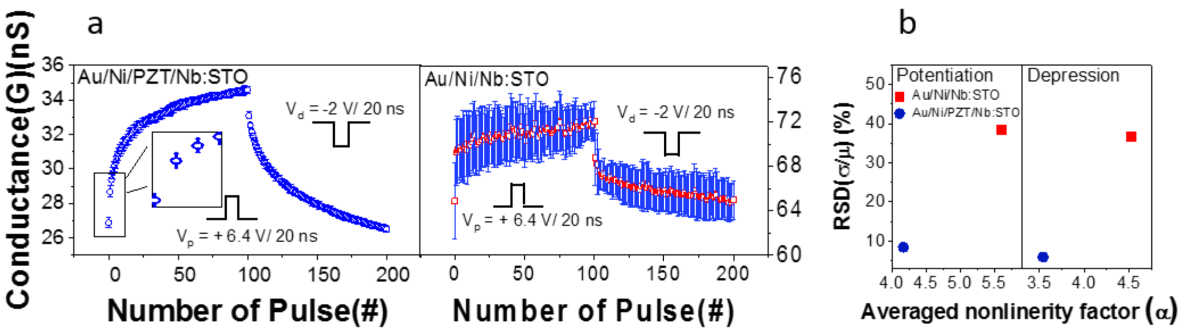

Figure S10. Analog synaptic behaviors obtained using an identical programming scheme for Ni/PZT/Nb:STO and Ni/Nb:STO devices.

We also measured analog weight updates under an ideltical programming scheme to discover the effect of the gradual change of current from the polarization modulation in ferroelectric. As shown in Figure S10a, same voltage pulses for potentiation (6.4 V amplitude and 20 ns pulse duration) and depression (-2 V amplitude and 20 ns duration) are applied to induce 100 conductance states in both the Ni/PZT/Nb:STO and Ni/Nb:STO devices. During 9 potentiation-depression cycles, the Ni/PZT/Nb:STO devices shows more linear and stable analog weight updates than the Ni/Nb:STO device. Figure S10b shows that the average value and RSD (σ/μ) for the nonlinearity factor of the Ni/PZT/Nb:STO device are 4.1 and 8.4 % for potentiation and 3.5 and 5.9 % for depression, respectively, whereas those of the Ni/Nb:STO device are 5.5 and 38.3 % for potentiation and 4.5 and 36.6 % for depression, respectively.

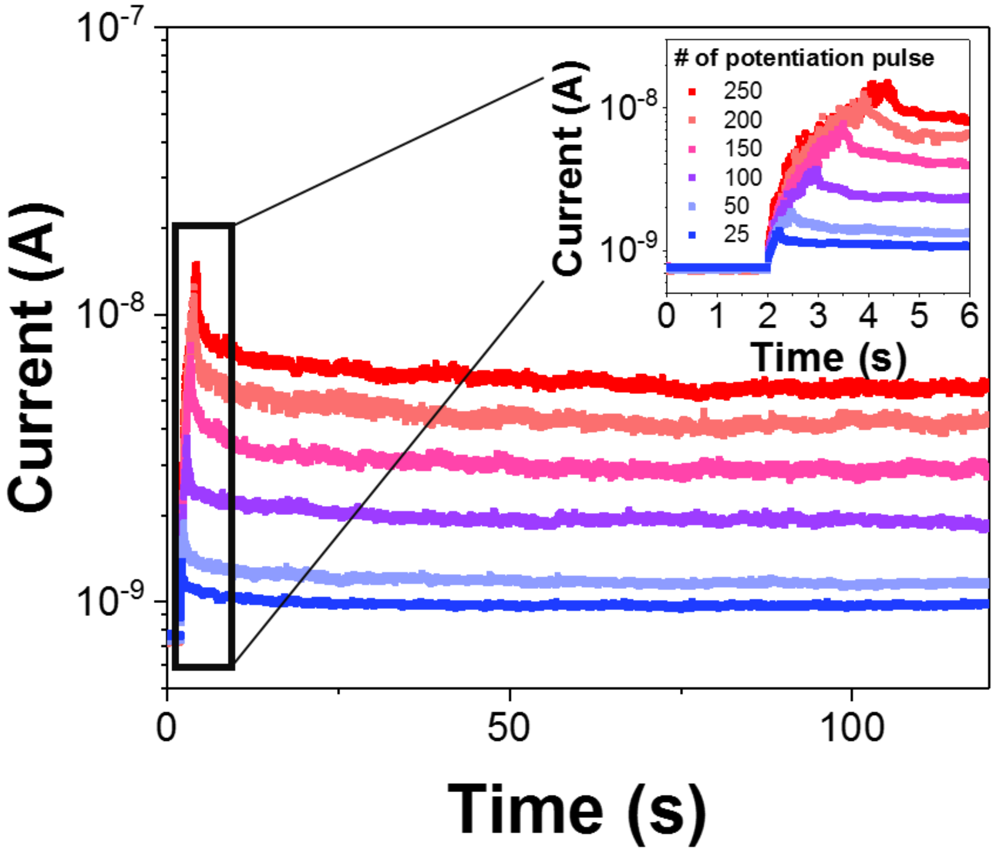

Figure S11. The long-term memory of multi-states obtained by different pulse numbers.

We measured current evolution to investigate the long-term memory of multi-states obtained by pulse trains with a fixed amplitude (6.4 V), a fixed duration (100 ns) and variable pulse numbers (25, 50, 100, 150, 200, and 250). The reading pulse with -0.1 V amplitude and 10 ms duration was used. A particular transition to a gradually higher current level is observed with repeated stimulation of input pulse, as shown in Fig. S11. A long-lived transition to a higher current level is achieved after the last input pulse, which corresponds to long-term memory. It is noticeable that multi-states obtained by different pulse numbers maintain distinguishable current levels during long time after the application of the pulse train.

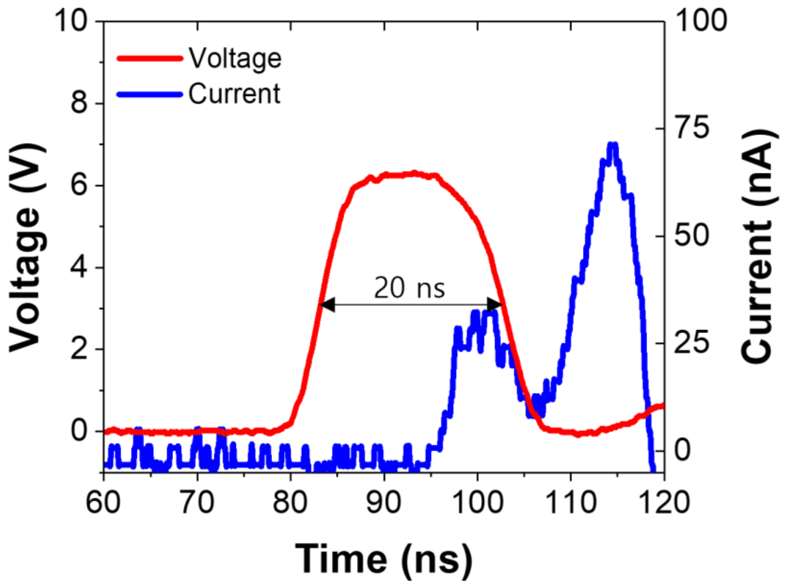

Figure S12. Ultrafast pulse measurement for the Ni/PZT/Nb:STO device.

To confirm that nanosecond pulses can induce conductance updates in our device, we conducted an ultrafast pulse measurement by using a function generator (Tektronix AFG3101) and an oscilloscope (Tektronix DPO5104B). A pulse generator delivers voltage pulses with an amplitude of 6.4 V and a duration of about 20 ns. The voltage pulse applied to the top electrode was recorded by the channel 1 of the oscilloscope while the signal transmitted through the device was simultaneously recorded by the channel 2. The write current was measured to be 71 nA, which resulted in the energy consumption of about 9 fJ calculated from the equation of E = V (pulse)×I (pulse) × t (pulse width).

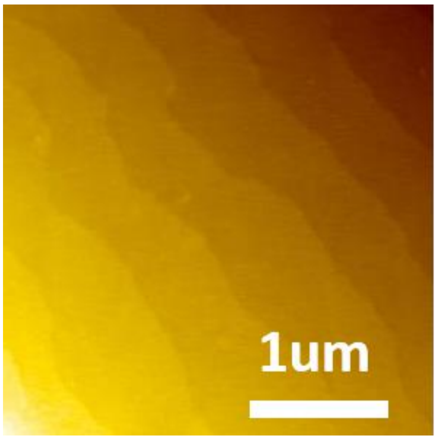

Figure S13. AFM topography image of the (001)-orientated Nb:STO substrate after chemical etching and annealing.

The Nb:STO substrates were etched using NH_4F buffered-HF solution and then annealed at 1000 °C for 1 h in air to form a TiO_2 single-terminated step-terrace surface. A well-organized terrace structure was identified in the AFM topography image, as shown in Figure S13. The step height is approximately 0.4 nm, which corresponds to the height of one unit cell of Nb:STO. This substrate was used for the epitaxial growth of the PZT thin film.[11]

References

[1] Skriver, L., Rosengaard, N. *Phys. Rev. B.* **1992**, 46, 7157.

[2] Mikheev, E., Hoskins, B., Strukov, D., Stemmer, S. *Nat. Commun*. **2014**, 5, 3990.

[3] Gao, Q., Chen, B., Yu, Q., Zhang X., Zhu, H. *J. Alloys Compd.* **2013**, 569, 62.

[4] Choi, S., Tan, S., Li1, Z., Kim, Y., Choi, C., Chen, P., Yeon, H., Yu, S., Kim, J. *Nat. Mater.* **2018**, 17, 335.

[5] Moon, K., Lim, S., Park, J., Sung, C., Oh, S., Woo, J., Lee, J., Hwang, H. *Faraday Discuss*. **2019**, 213, 421.

[6] Xi, Z., Ruan, J., Li, C., Zheng, C., Wen, Z., Dai, J., Li, A., Wu, D. *Nat. Commun.* **2017**, 8, 15217.

[7] Kuzum, D., Yu, S., Wong, H. *Nanotechnology* **2013**, 24, 382001.

[8] Li, J., Ge, C., Du, J., Wang, C., Yang, G., Jin, K. *Adv. Mater.* **2020**, 32, 1905764.

[9] Chen, P., Peng, X., Yu, S. I*EEE Trans. Comput. Des. Integr. Circuits Syst.* **2018**, 37, 3067.

[10] Choi, S., Yang, J., Wang, G. *Adv. Mater*. **2020**, 32, 2004659.

[11] Li, Y., Sun, J., Zhao, J., Shen, B. J. *Appl. Phys*. **2013**, 114, 154303.
